# Thermal processing methods differentially affect the protein quality of Chickpea (*Cicer arietinum*)

**DOI:** 10.1002/fsn3.1597

**Published:** 2020-05-12

**Authors:** Matthew G. Nosworthy, Gerardo Medina, Adam J. Franczyk, Jason Neufeld, Paulyn Appah, Alphonsus Utioh, Peter Frohlich, Bunyamin Tar'an, James D. House

**Affiliations:** ^1^ Department of Food and Human Nutritional Sciences University of Manitoba Winnipeg MB Canada; ^2^ Food Development Centre Portage la Prairie MB Canada; ^3^ Canadian International Grains Institute Winnipeg MB Canada; ^4^ College of Agriculture and Bioresources University of Saskatchewan Saskatoon SK Canada; ^5^ Richardson Centre for Functional Foods and Nutraceuticals University of Manitoba Winnipeg MB Canada; ^6^ Canadian Centre for Agri‐Food Research in Health and Medicine University of Manitoba Winnipeg MB Canada; ^7^ Department of Animal Science University of Manitoba Winnipeg MB Canada

**Keywords:** cultivar, digestible indispensable amino acid score, processing, protein digestibility‐corrected amino acid score, protein efficiency ratio

## Abstract

Chickpea is a widely produced pulse crop, but requires processing prior to human consumption. Protein bioavailability and amino acid quantity of chickpea flour can be altered by multiple factors including processing method. For this reason, the protein quality of processed chickpea flour was determined using in vivo and in vitro analyses for processed chickpeas. Processing differentially affected the protein digestibility‐corrected amino acid score (PDCAAS) of chickpeas with extruded chickpea (83.8) having a higher PDCAAS score than both cooked (75.2) and baked (80.03). Interestingly, the digestible indispensable amino acid score (DIAAS) value of baked chickpea (0.84) was higher compared to both extruded (0.82) and cooked (0.78). The protein efficiency ratio, another measure of protein quality, was significantly higher for extruded chickpea than baked chickpea (*p* < .01). I*n vivo* and in vitro analysis of protein quality were well correlated (*R* ^2^ = .9339). These results demonstrated that under certain circumstances in vitro methods could replace the use of animals to determine protein quality.

## Graphical abstract

In this article, chickpeas were processed using three different methods and the resulting impact on protein quality measured. Protein quality was also assessed through multiple metrics (PER/PDCAAS/DIAAS) with in vitro and in vivo methods being compared, where appropriate. This work contributes to the body of knowledge relating to the nutritional quality, and potential health impacts, of chickpea processing method selection

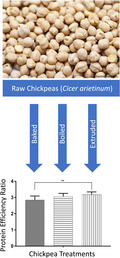

## 1 INTRODUCTION

Chickpeas (*Cicer arietinum* L.) are the second most produced pulse crop worldwide with 13.7 MT in 2014, falling between dry beans (27.6 MT) and field peas (12.5 MT) (FAOSTAT, 2017). While chickpeas are grown in more than fifty countries, the major chickpea‐producing countries are India, Australia, and Myanmar. Chickpeas are a good source of protein and carbohydrates in comparison with other pulses (Tavano, da Silva Jr, Demonte, & Neves, 2008), as well as being a good source of other nutrients such as minerals, fiber, and vitamins. Previous work has shown chickpeas to be limiting in sulfur amino acids, compared to human nutritional requirements, but have also been found to be lower in valine (Clemente, Sánchez‐Vioque, Vioque, Bautista, & Millán, 1998; El‐Adawy, 2002). This lower amino acid content, in conjunction with reduced protein digestibility compared to other protein sources, has been implicated as the main reason for the lower nutritional value of chickpea protein (Mudryj, Yu, & Aukema, 2014). Anti‐nutritive factors such as trypsin inhibitors, protease inhibitors, and lectins can alter amino acid bioavailability by limiting protein digestibility (Tavano et al., 2008). Preparatory methods such as extrusion, baking, or cooking can alter the concentration and/or activity of these anti‐nutritive factors and may thereby alter the bioavailability and digestibility of amino acids and protein, respectively, in chickpea flours.

The effect of extrusion on the protein or amino acid content of chickpeas has not been well studied; however, there has been thorough investigation regarding extruded bean products (Al‐Marzooqi & Wiseman, 2009; Arija et al., 2006; Batista Prudencio, & Fernandes, 2010; Coffey, Uebersax, Hosfield, & Bennink, 1993; Kelkar et al., 2012; Simons et al., 2015). This processing method is widely used in commercial production of snacks and is capable of reducing the activity of anti‐nutritive compounds (Al‐Marzooqi & Wiseman, 2009; Batista et al., 2010; Coffey et al., 1993; Kelkar et al., 2012; Simons et al., 2015). The reduction of anti‐nutritive activity/concentration is also found after cooking (Wang, Hatcher, & Gawalko, 2008; Wang, Hatcher, Toews, & Gawalko, 2009; Wang, Hatcher, Tyler, Toews, & Gawalko, 2010) and autoclaving, an experimental surrogate for baking (Marquardt, Campbell, Stothers, & Mckirdy, 1974; Umoren, Tewe, & Bokanga, 1997). While food preparation method may increase protein digestibility, it can also modify protein content and amino acid composition (Arija et al., 2006; Batista et al., 2010; Candela, Astiasaran, & Belli, 1997; Fernández, López‐Jurado, Aranda, & Urbano, 1996; Simons et al., 2015; Wang et al., 2010). Extrusion does not alter protein content in beans (Batista et al., 2010; Simons et al., 2015), but has been shown to reduce the content of both cysteine and methionine, potentially due to the disruptive forces of the extruding process as well as the high temperatures used in extrusion (Arija et al., 2006). Cooking, on the other hand, has been shown to result in higher protein content in kidney beans, chickpeas, and faba beans (Candela et al., 1997; Fernández et al., 1996; Wang et al., 2010) due to carbohydrate loss (Savage &Thompson, 1993; Verde, Frias, & Verde, 1992), while also increasing the concentration of essential amino acids (Alajaji & El‐Adawy, 2006; Khattab, Arntfield, & Nyachoti, 2009). Autoclaving flours resulted in reduced available lysine content. However, protein utilization was increased due to reduced anti‐nutritive factor concentration/activity (del Cueto, Martinez, & Frampton, 1960; Srihara & Alexander, 1983).

Protein quality can be determined by multiple methods including protein efficiency ratio (PER), protein digestibility‐corrected amino acid score (PDCAAS), and digestible indispensable amino acid score (DIAAS). PER is a measurement of growth mandated for use in the regulation of Canadian protein content claims (Health Canada, 1981). In the United States, PDCAAS is the required method of determining protein quality for claim purposes (FAO/WHO, 1991) while the most recently developed method for measuring protein quality, DIAAS, is not used for regulatory purposes in any jurisdiction (FAO/WHO, 2013). One aspect of the current study was to determine the effects of extrusion, baking, and cooking (boiling) on the protein quality of chickpeas. Protein digestibility was also determined via in vitro methodology for the calculation of in vitro PDCAAS, which was used to investigate the correlation between in vivo and in vitro methods of protein quality assessment (Nosworthy, Franczyk, et al., 2017). This study also afforded an opportunity to investigate the potential for cultivar or growing location to impact protein content and amino acid composition of Canadian chickpeas, as had been previously demonstrated in India (Singh, Kumar, & Gowda, 1983).

## 2 MATERIALS AND METHODS

### 2.1 Statement on animal ethics

All animal procedures received approval by the University of Manitoba's Institutional Animal Care Committee, which utilize the appropriate guidelines established by the Canadian Council on Animal Care (CCAC, 2017).

### 2.2 Chemicals

Formic acid (88% ACS), hydrogen peroxide (30%), orthophosphoric acid (85%), and glacial acetic acid were purchased from Fisher. Barium hydroxide (>98%), 1,1,1‐trichloro‐2‐methyl‐2‐propanol (98%), and ethanolamine (>99%) were purchased from Sigma.

### 2.3 Sample procurement and preparation

Samples of chickpeas for processing were provided by Saskcan Pulse Trading, Thompsons Ltd., and Viterra. Chickpeas from different suppliers were combined and thoroughly mixed before processing. Milling of the combined samples to generate flour for extrusion and baking was performed milled on a hammer mill using a 0.050 inch screen (Jacobson 120‐B hammer mill) (Nosworthy, Franczyk, et al., 2017). The extrusion and baking of the chickpea flour, as well as the cooking of the chickpeas, were performed as previously described (Nosworthy et al., 2018). Baked samples underwent hammer milling (Fitz mill—model #D comminutor VHP‐506‐55B), with screen hole size of 0.020 inch, round, followed by a 20 mesh screening on a sifter (Kason, Vibro Screen, K24 3 SS). Extruded and cooked samples were hammer‐milled (Jacobson 120‐B hammer mill), with screen hole size of 0.050 inch.

Samples of the chickpea cultivars CDC Frontier, CDC Leader, and CDC Orion grown at the locations Cabri, Limerick, and Moose Jaw in Saskatchewan in 2014 were provided for analysis by Bunyamin Tar'an, University of Saskatchewan. CDC Frontier is a medium‐seeded kabuli; while CDC Leader and CDC Orion are large‐seeded kabuli type.

### 2.4 Sample analysis

Percent crude protein (CP; *N* × 6.25) was determined via Dumas Nitrogen Analyzer (Dumatherm DT, Gerhardt Analytical Systems), while percent dry matter (DM) and ash were determined according to AOAC guidelines (AOAC, 1995). The selection of a Jones factor of 6.25 was done according to recommendations for the determination of protein quality (AOAC, 1995). A control sample (NIST 3234, National Institute of Standards and Technology) was included in each amino acid assay to ensure the accuracy of the assay. Percent crude fat was determined by hexane extraction and gravimetrics (AOAC 2003.06). Sulfur amino acid content was determined according to AOAC 994.12 with the remaining amino acids, excepting tryptophan, determined according to AOAC 982.30. Analysis of tryptophan was performed as previously described (ISO, 2005; Nosworthy, Franczyk, et al., 2017).

### 2.5 Protein quality assessment

PDCAAS, in vitro PDCAAS, DIAAS, and PER of processed chickpeas were determined as previously described (House, Neufeld, & Lesson, 2010; Nosworthy et al., 2018; Tinus, Damour, Van Riel, & Sopade, 2012).

### 2.6 Statistics

True fecal protein digestibility (TFPD) and PER results (*n* = 10) were compared via one‐way ANOVA with Tukey's selected as the post hoc test. Correlations between both in vivo and in vitro digestibilities, and PDCAAS/in vitro PDCCAS (*n* = 4) were determined via regression analysis (GraphPad Prism, 7.0, GraphPad Software).

## 3 RESULTS AND DISCUSSION

### 3.1 Proximate analysis

Sample proximate data are presented in Table 1, with crude fat/protein and amino acid composition being presented on a DM basis. While the dry matter of the unprocessed chickpea flour (91.95%) was lower than that of any processed flours, it was similar to previously reported results (92.32%) (Canadian Nutrient File, 2015). There was little difference between the dry matter of the processed flours, ranging from 95.57% after extrusion to 97.68% after cooking and freeze drying. The fat content of the untreated chickpeas was higher than previously reported (6.63% vs. approximately 5.0%–6.0%) (Canadian Nutrient File, 2015; Jukanti, Gaur, Gowda, & Chibbar, 2012; Tavano et al., 2008; Wang & Daun, 2004), and all processing methods increased the fat content by 0.57% (extrusion), 1.14% (cooking), and 3.28% (baking). Protein content of untreated chickpeas was 19.93%, protein content of cooked chickpeas being the highest at 22.10%, with extrusion being 21.15%, and baking 20.89%, similar to previous results (Canadian Nutrient File, 2015; FAO/WHO, 1991; Jukanti et al., 2012; Nosworthy, Neufeld, et al., 2017; Tavano et al., 2008; Wang & Daun, 2004). While cooking did increase the protein content to a greater extent than the other processing methods, from 19.93% to 22.10% as previously reported (Candela et al., 1997; Fernández et al., 1996; Wang et al., 2009), no processing method dramatically altered the chickpea protein content.

**TABLE 1:** Proximate analysis and amino acid composition of untreated, extruded, cooked, and baked chickpea flour presented on a dry matter basis

	%DM^a^	%CF^b^	%CP^c^	ASP^d^	THR	SER	GLU	PRO	GLY	ALA	CYS	VAL	MET	ILE	LEU	TYR	PHE	HIS	LYS	ARG	TRP
Casein	93.56	0.21	92.43	8.32	3.58	6.03	21.43	10.44	1.44	3.38	0.83	5.37	1.55	4.10	8.97	5.16	4.91	2.93	7.44	3.33	1.15
Chickpea																					
Untreated	91.95	6.63	19.93	2.22	0.64	1.01	2.98	0.64	0.65	0.87	0.28	0.77	0.28	0.69	1.38	0.45	1.07	0.52	1.19	1.62	0.24
Extruded	95.57	7.20	21.15	2.75	0.80	1.25	3.70	0.82	0.81	1.07	0.28	0.96	0.26	0.89	1.75	0.53	1.29	0.64	1.45	1.90	0.23
Cooked	97.68	7.77	22.10	2.76	0.79	1.26	3.56	0.75	0.81	1.06	0.28	1.00	0.26	0.90	1.79	0.55	1.32	0.66	1.48	1.97	0.20
Baked	96.67	9.91	20.89	2.62	0.76	1.21	3.59	0.81	0.79	1.04	0.29	0.92	0.28	0.85	1.68	0.56	1.28	0.64	1.33	1.80	0.22

Note

Values are expressed as % by weight (dry matter basis).

^a^ DM = dry matter content.

^b^ CF = crude fat, determined by hexane extraction.

^c^ CP = crude protein = nitrogen content (determined by DUMAS analysis) x 6.25.

^d^ Amino acid abbreviations: ASP, aspartate; THR, threonine; SER, serine; GLU, glutamate; PRO, proline; GLY, glycine; ALA, alanine; CYS, cysteine; VAL, valine; MET, methionine; ILE, isoleucine; LEU, leucine; TYR, tyrosine; PHE, phenylalanine; HIS, histidine; LYS, lysine; ARG, arginine; TRP, tryptophan.

### 3.2 Amino acid score and protein digestibility

Sample amino acid composition is presented in Table 1, and amino acid scores are presented in Table 2. The first limiting amino acid in all processed chickpea flours was tryptophan. While this agrees with certain studies (Nosworthy, Franczyk, et al., 2017; Tavano et al., 2008), others have found that chickpeas were initially limited in the sulfur containing amino acids, cysteine, and methionine (Jukanti et al., 2012; Wang & Daun, 2004). The amino acid scores of the extruded (0.97) cooked (0.86) and baked (0.95) chickpeas were higher than anticipated. Compared to previous work in chickpeas, which found amino acid scores of 0.61–0.62, the chickpeas used in this study have a different composition that is more similar to the human nutritional pattern for children aged 2–5 years put forth by the FAO/WHO (1991), resulting in a higher amino acid score (FAO/WHO, 1991). It is also worth noting that while chickpeas can be limiting in sulfur amino acids, the amino acid score for methionine + cysteine is either the same, 1.03 for extruded flours, or greater, 1.07 for baked flours, than that found in casein (1.03). These high amino acid scores for chickpea sulfur amino acids have been corroborated by similar findings in other chickpea samples (Bai, Nosworthy, House, & Nickerson, 2018), and the fidelity of the amino acid protocol has been confirmed via the use of standards and the analysis of a control sample, soy flour. The difference in amino acid composition and the resulting amino acid scores of these processed chickpeas could be due to the differences in varieties, crop growing location, or other environmental factors. Determining how agronomy can influence amino acid quantity could potentially result in higher quality proteins from plant‐based sources.

**TABLE 2:** Amino Acid Scores of casein and extruded, cooked and baked chickpea flour

	THR^a^	VAL	MET + CYS	ILE	LEU	PHE + TYR	HIS	LYS	TRP
Casein	1.14	1.66	**1.03**	1.59	1.47	1.73	1.67	1.39	1.13
Chickpea									
Extruded	1.10	1.30	1.03	1.51	1.25	1.37	1.58	1.19	**0.97**
Cooked	1.05	1.29	0.97	1.45	1.23	1.35	1.55	1.15	**0.86**
Baked	1.07	1.26	1.07	1.44	1.22	1.40	1.61	1.10	**0.95**

Note

Bolded values indicate the first limiting amino acid. The reference pattern used to calculate the amino acid scores was as follows (mg/g protein): Thr – 34, Val −35, Met + Cys – 25, Ile – 28, Leu – 66, Phe + Tyr – 63, His – 19, Lys – 58, Trp – 11.

^a^ Amino acid abbreviations: THR, threonine; VAL, valine; CYS, cysteine; MET, methionine; ILE, isoleucine; LEU, leucine; PHE, phenylalanine; TYR, tyrosine; HIS, histidine; LYS, lysine; TRP, tryptophan.

Protein digestibility values as determined by in vitro and in vivo measurement are presented in Table 3. Chickpea TFPD significantly differed between cooked (87.17%) and baked (84.62%; *p* < .05). No difference was detected among cooked, baked, and extruded (86.56%) samples. While these digestibilities are higher than reported for heated chickpea flour, 78.75% (Tavano et al., 2008), the cooked true protein digestibility is similar to that found in another study, 85.02% (Nosworthy, Neufeld, et al., 2017). The chickpea protein digestibilities found in this study are similar to previous findings in canned chickpeas, 88%–89% (FAO/WHO, 1991). The digestibility of raw chickpeas, as determined in vitro, has been reported as between 34%–76% (Jukanti et al., 2012), with one study determining a protein digestibility of 89.01%, which increased to 96.94% after heating (Monsoor &Yusuf, 2002). This variability in in vitro digestibilities could be attributed to different methods of analysis as methods can differ in number/type of digestive enzymes, pH, and incubation time, all of which can alter the final value attributed to protein digestibility. In this study, in vivo and in vitro protein digestibility values differed in that while baked chickpea had the lowest digestibility in vivo and in vitro, cooked chickpea had the highest in vivo digestibility, while extruded chickpea had the highest digestibility in vitro. For cooked and baked chickpeas, in vitro digestibility was lower than in vivo, while the in vitro digestibility was greater than in vivo for extruded chickpeas. As the in vitro method used in this study incorporates a limited representation of the digestive process compared to an in vivo system, it is unsurprising that this in vitro system would not perfectly mimic the digestive process.

**TABLE 3:** Adjusted protein efficiency ratio, protein digestibility‐corrected amino acid scores and in vitro protein digestibility‐corrected amino acid scores of extruded, cooked, and baked chickpea flour

	Adj. PER^a^	AAS^b^	TFPD^c^	IVPD^d^	PDCAAS^e^	IVPDCAAS^f^
Casein	2.50	1.03	97.3 (0.61)	90.7 (2.52)	100	93.5
Chickpea						
Extruded	2.56	0.97	86.6 (1.0)^AB^	87.1 (0.09)	83.8	84.3
Cooked	2.47	0.86	87.1 (2.5)^B^	83.5 (3.53)	75.2	72.0
Baked	2.30	0.95	84.6 (2.0)^A^	80.4 (0.63)	80.0	76.0

Note

Numbers in parentheses indicate *SD* where applicable. TFPD was analyzed via one‐way ANOVA with Tukey's post hoc test. Superscripts with different letters are significantly different. PDCAAS is calculated as the product of AAS and TFPD while IVPDCAAS is the product of AAS and IVPD.

^a^ Adj. PER = adjusted protein efficiency ratio (against casein set to 2.50).

^b^ AAS = amino acid score.

^c^ %TFPD = % true fecal protein digestibility.

^d^ IVPD = in vitro protein digestibility.

^e^ PDCAAS = protein digestibility‐corrected amino acid score.

^f^ IVPDCAAS = in vitro protein digestibility‐corrected amino acid score. *n* = 10 for Adj. PER and TFPD; *n* = 2 for IVPD and *n* = 1 for AAS, PDCAAS, IVPDCAAS.

### 3.3 PDCAAS and in vitro PDCAAS

The protein digestibility‐corrected amino acid score (PDCAAS) and in vitro PDCAAS score are presented in Table 3. The PDCAAS of processed chickpeas was 75.20% for cooked, 80.01% for baked, and 83.80% for extruded. Previously, the PDCAAS value for cooked chickpeas was determined to be 51.9%35, and 44.1% for heated chickpea flour (Tavano et al., 2008). These lower PDCAAS values were primarily due to lower amino acid scores (0.61 and 0.62) than that determined in the chickpeas used in this study (0.86–0.97). The in vitro PDCAAS values shared a similar pattern to those determined in vivo, with cooking having the lowest value (72.02%), followed by baked (76.04%) and extruded (84.33%). The protein content and PDCAAS values determined for baked, cooked, and extruded chickpeas in this study would support an “Excellent Source” claim, as the corrected protein content for these products would average 15 g for a 90 g RACC.

**FIGURE 1:**
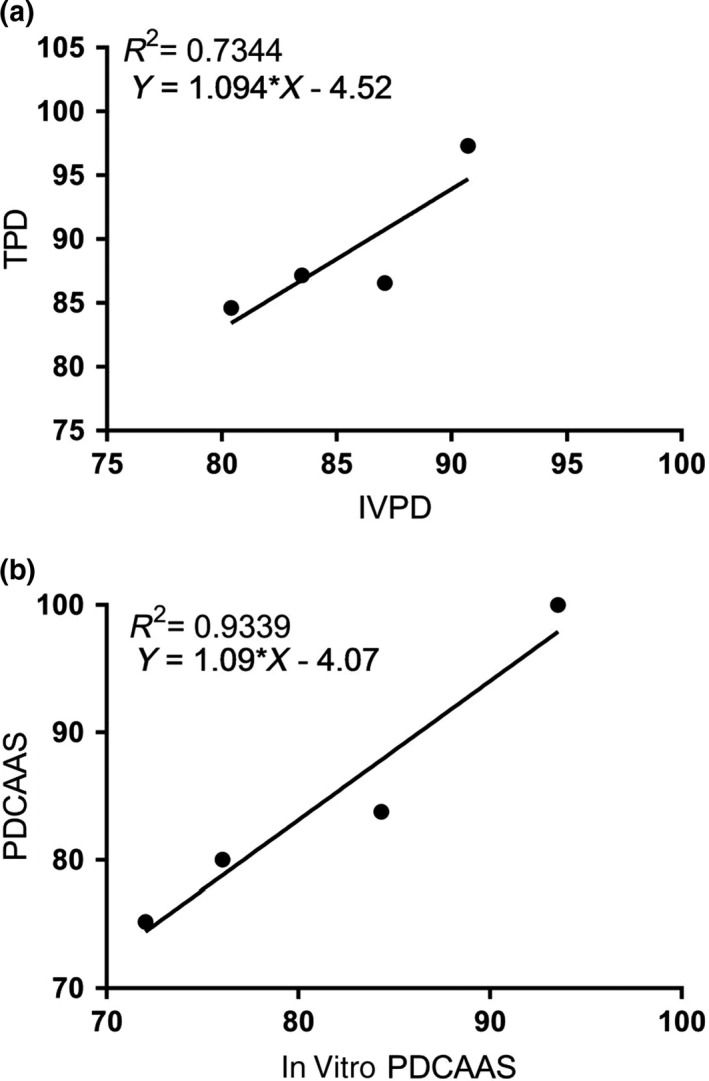
Relationship between the digestibility extruded, cooked, and baked chickpea flour determined by in vitro and in vivo methods (a) and the relationship between the protein digestibility‐corrected amino acid scores calculated using in vitro and in vivo digestibilities (b). IVPD, in vitro protein digestibility; IVPDCAAS, in vitro protein digestibility‐corrected amino acid score; PDCAAS, protein digestibility‐corrected amino acid score; TPD, true protein digestibility

Protein quality assessment requires animal experimentation to quantify protein digestibility (FAO/WHO, 1991). However, companies and consumers desire alternatives to animal experimentation wherever possible. It has been demonstrated that i*n vitro* protein digestion could provide an effective alternative to in vivo analysis (Nosworthy & House, 2017; Tavano, Neves, & da Silva, 2016). This study used a one‐step pH drop method (Tinus et al., 2012), and for determining in vitro protein digestion for comparison with that found in the rodent model. The correlation between in vitro and in vivo digestibility was *R* ^2^ = .7344, while the correlation between PDCAAS and in vitro PDCAAS had an *R* ^2^ value of .9339 (*p* = .0336). This relationship between in vivo and in vitro protein quality is similar to that found in other plant‐based protein sources (Nosworthy, Franczyk, et al., 2017; Nosworthy & House, 2017; Tavano et al., 2016), further supporting the concept of using in vitro PDCAAS as a surrogate for animal experimentation (Figure 1).

### 3.4 DIAAS

The digestible indispensable amino acid score (DIAAS) was recommended in 2013 by the FAO/WHO as a PDCAAS replacement (FAO/WHO, 2013). DIAAS differs from PDCAAS in that it should use ileal amino acid digestibility, not TFPD, and consider amino acids as nutrients rather than protein itself (FAO/WHO, 1991, 2013). As the amount of data on ileal digestibility of amino acids is limited, this study used fecal protein digestibility in the calculation of DIAAS, as per recommendations (FAO/WHO, 2013). The chickpea DIAAS data are presented in Table 4. The DIAAS value for baked chickpea (0.84) was higher than cooked (0.78) and extruded (0.82), and for all processing methods, sulfur amino acids were limiting. Previous work found a DIAAS value of 0.67 for cooked chickpeas (Nosworthy, Neufeld, et al., 2017), whereas in this study the DIAAS value for cooked chickpea was 0.78. When compared to PDCAAS, DIAAS values for baked and cooked were higher, while the DIAAS value for extruded chickpea was lower. This might be explained as the reference pattern used in the determination of DIAAS and PDCAAS is different; specifically, the requirements for sulfur amino acids were lowered to 25 mg/g protein for DIAAS from 27 mg/g protein for PDCAAS, and the tryptophan requirement from was reduced from 11 mg/g (PDCAAS) to 8.5 mg/g (DIAAS).

**TABLE 4:** Digestible Indispensable Amino Acid values of extruded, cooked, and baked chickpea flour

	THR^a^	VAL	MET + CYS	ILE	LEU	PHE + TYR	HIS	LYS	TRP	DIAAS^b^
Casein	1.22	1.31	**0.93**	1.35	1.43	2.04	1.54	1.37	1.42	0.93
Chickpea										
Extruded	1.04	0.91	**0.82**	1.14	1.08	1.43	1.30	1.05	1.08	0.82
Cooked	1.01	0.92	**0.78**	1.11	1.07	1.42	1.28	1.02	0.97	0.78
Baked	0.99	0.87	**0.84**	1.07	1.03	1.43	1.30	0.94	1.04	0.84

Note

Bolded values reflect first limiting amino acid.

^a^ Amino acid abbreviations: THR, threonine; VAL, valine; CYS, cysteine; MET, methionine; ILE, isoleucine; LEU, leucine; PHE, phenylalanine; TYR, tyrosine; HIS, histidine; LYS, lysine; TRP, tryptophan.

^b^ DIAAS = digestible indispensable amino acid score. DIAAS was calculated using true protein digestibility.

### 3.5 PER

**FIGURE 2:**
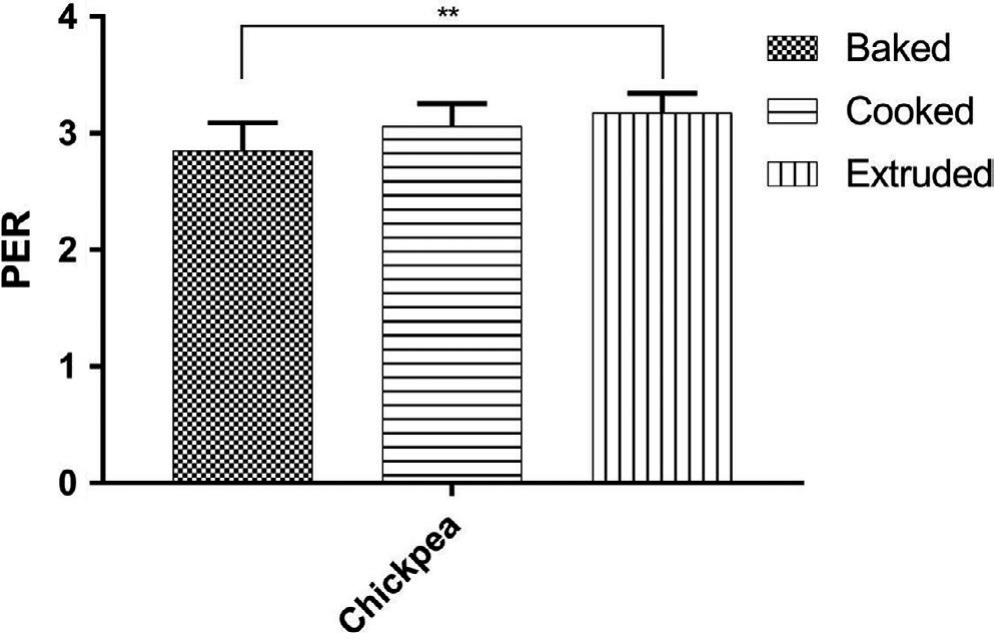
Protein efficiency ratio (PER) values of extruded, cooked, and baked chickpea flour. Hatched bars indicate baked flour, horizontal bars are cooked flour, and vertical bars are extruded flour. Mean ± *SD* (*n* = 10). Data were analyzed via one‐way ANOVA with Tukey's post hoc test. **p < .01

Compared to PDCAAS and DIAAS, the protein efficiency ratio (PER) is a growth measurement comparing weight gain over a period of 28 days to the amount of protein consumed. Currently, Health Canada mandates the use of PER as a protein quality measurement to regulate content claims for protein (Health Canada, 1981). The chickpea PER data are presented in Figure 2. Extruded chickpea had a significantly higher PER than baked (*p* < .01); however, no significant difference was found between either extruded and cooked or cooked and baked chickpeas. A study investigating baked chickpeas determined a PER of 2.88, compared to 2.3 in this study while previous work on cooked chickpeas determined a PER of 2.32 versus 2.42 in this study (Nosworthy, Franczyk, et al., 2017; Tavano et al., 2008). To account for measurement variability, PER values are also adjusted to the relative PER for the control, casein, which is set to 2.5 (Health Canada, 1981). These values, presented in Table 3, indicate that for chickpeas, extrusion resulted in the highest growth rate based on protein consumption, followed by cooking and baking.

### 3.6 Effects of cultivar and location on proximate and amino acid composition of chickpeas

The proximate and amino acid composition of three chickpea cultivars (Frontier, Leader, and Orion) grown at three locations in Saskatchewan (Cabri, Limerick, Moose Jaw) in 2014 are presented in Table 5 with the resulting amino acid scores presented in Table 6. Broadly, the varietal Orion had a lower protein content (18.11%) than either Frontier (20.05%) or Leader (20.56%). Similarly, one location, Limerick, was found to generate a lower protein content on average (17.67%) than Cabri (20.85%) or Moosejaw (20.20%). Limerick is known as a drier chickpea growing area in Saskatchewan compared to Moose Jaw and Cabri which may have resulted in lower total protein in Limerick. However, this is based on a small sample size and that variation is within the normal range found in this study for processed chickpeas as well as chickpea protein content reported elsewhere (Canadian Nutrient File, 2015; FAO/WHO, 1991; Jukanti et al., 2012; Nosworthy, Neufeld, et al., 2017; Tavano et al., 2008; Wang & Daun, 2004). The amino acid scores ranged from a low of 0.77 for valine (Leader grown in Cabri) to 0.92 (Leader grown in Limerick). The variation of amino acid scores within cultivars across location suggests that location of growth is as important as cultivar selection, which agrees with the findings of Singh et al. (1983), although their focus was protein content not amino acid composition specifically. Given that the samples were available for only one growing season, caution should be used in extrapolating the current data to definitive varietal or location differences. These should ideally be assessed across multiple cropping years. However, the current data do highlight the potential for shifts in the amino acid pattern of Canadian‐grown chickpeas.

**TABLE 5:** Proximate analysis and amino acid composition of chickpea varietals grown in different locations in Saskatchewan presented on a dry matter basis

Location	Varietal	%DM^a^	%CF^b^	%CP^c^	ASP^d^	THR	SER	GLU	PRO	GLY	ALA	CYS	VAL	MET	ILE	LEU	TYR	PHE	HIS	LYS	ARG	TRP
Cabri	Frontier	92.50	5.82	20.47	2.07	0.67	0.98	3.03	0.74	0.62	0.68	0.30	0.62	0.30	0.64	1.36	0.55	1.08	0.40	1.25	1.44	0.19
Cabri	Leader	92.88	6.12	22.61	2.11	0.68	1.00	3.07	0.76	0.63	0.68	0.30	0.61	0.31	0.63	1.37	0.53	1.06	0.38	1.31	1.43	0.20
Cabri	Orion	92.08	6.65	19.48	1.90	0.61	0.87	2.74	0.67	0.54	0.61	0.29	0.54	0.29	0.57	1.21	0.51	0.94	0.36	1.09	1.23	0.18
Limerick	Frontier	92.31	6.41	18.35	1.86	0.62	0.87	2.69	0.67	0.57	0.62	0.27	0.58	0.29	0.59	1.23	0.51	0.96	0.36	1.15	1.26	0.19
Limerick	Leader	92.45	6.59	18.32	1.77	0.59	0.80	2.56	0.63	0.53	0.57	0.27	0.61	0.27	0.63	1.19	0.48	0.91	0.35	1.14	1.14	0.19
Limerick	Orion	92.62	7.52	16.33	1.60	0.52	0.74	2.30	0.58	0.47	0.52	0.25	0.46	0.26	0.50	1.02	0.43	0.78	0.31	1.03	0.98	0.17
Moosejaw	Frontier	92.28	5.97	21.34	2.05	0.62	0.94	2.98	0.72	0.58	0.65	0.28	0.58	0.31	0.60	1.32	0.49	1.03	0.39	1.24	1.41	0.20
Moosejaw	Leader	93.04	6.36	20.76	2.04	0.63	0.95	2.95	0.72	0.58	0.64	0.28	0.59	0.29	0.63	1.32	0.53	1.04	0.40	1.25	1.42	0.20
Moosejaw	Orion	92.82	6.96	18.52	1.93	0.62	0.89	2.82	0.69	0.56	0.61	0.31	0.62	0.32	0.64	1.28	0.51	1.00	0.41	1.22	1.32	0.18

Note

Values are expressed as % by weight (dry matter basis).

^a^ DM = dry matter content.

^b^ CF = crude fat, determined by hexane extraction.

^c^ CP = crude protein = nitrogen content (determined by DUMAS analysis) x 6.25.

^d^ Amino acid abbreviations: ASP, aspartate; THR, threonine; SER, serine; GLU, glutamate; PRO, proline; GLY, glycine; ALA, alanine; CYS, cysteine; VAL, valine; MET, methionine; ILE, isoleucine; LEU, leucine; TYR, tyrosine; PHE, phenylalanine; HIS, histidine; LYS, lysine; ARG, arginine; TRP, tryptophan.

**TABLE 6:** Amino acid scores of chickpea varietals grown in different locations

Location	Varietal	THR^a^	VAL	MET + CYS	ILE	LEU	PHE + TYR	HIS	LYS	TRP
Cabri	Frontier	0.96	0.87	1.17	1.12	1.00	1.27	1.03	1.05	**0.85**
Cabri	Leader	0.88	**0.77**	1.08	1.00	0.92	1.12	0.90	1.00	0.81
Cabri	Orion	0.91	**0.79**	1.19	1.05	0.94	1.18	0.97	0.96	0.84
Limerick	Frontier	1.00	**0.90**	1.23	1.15	1.01	1.27	1.03	1.08	0.92
Limerick	Leader	0.95	0.95	1.19	1.23	0.98	1.20	1.02	1.08	**0.92**
Limerick	Orion	0.93	**0.81**	1.26	1.10	0.94	1.18	1.01	1.09	0.94
Moosejaw	Frontier	0.86	**0.78**	1.12	1.01	0.94	1.13	0.97	1.00	0.85
Moosejaw	Leader	0.90	**0.81**	1.10	1.08	0.97	1.20	1.03	1.04	0.88
Moosejaw	Orion	0.98	0.96	1.36	1.24	1.04	1.30	1.16	1.14	**0.89**

Note

Bolded values indicate the first limiting amino acid. The reference pattern used to calculate the amino acid scores was as follows (mg/g protein): Thr – 34, Val − 35, Met + Cys – 25, Ile – 28, Leu – 66, Phe + Tyr – 63, His – 19, Lys – 58, Trp – 11.

^a^ Amino acid abbreviations: THR, threonine; VAL, valine; CYS, cysteine; MET, methionine; ILE, isoleucine; LEU, leucine; PHE, phenylalanine; TYR, tyrosine; HIS, histidine; LYS, lysine; TRP, tryptophan.

## 4 CONCLUSION

In summary, processing is capable of altering protein quality through changes in either the amino acid composition or protein digestibility. This study has demonstrated that extrusion is the optimal method for producing the product with highest protein quality, while for home preparation baking chickpeas would provide a higher protein quality than cooking. The method of determining in vitro protein digestibility used in this study resulted in a good correlation between in vivo and in vitro measurements of PDCAAS, providing more support to the use of in vitro methods for determining protein quality. An overview of protein content and amino acid composition for three chickpea cultivars also revealed potential variation between protein content and amino acid scores depending on growing locations, suggesting that further study of the effects of environment x genetic interaction on protein content and quality can be pursued.

## CONFLICT OF INTEREST

The authors declare no conflicts of interest with regard to the described research, the publication of results, or financial issues. Funding for the study was provided via Agriculture and Agri‐Food Canada Growing Forward 2—Pulse Science Cluster program funded this project. M.G.N was also supported by funds received from the Global Institute for Food Security, University of Saskatchewan, Saskatoon, SK. MGN and JDH designed and oversaw the experimental components of the study, and MGN prepared the first draft of the manuscript. GM, JN, AF, PA, AU, PF, and MGN conducted the technical aspects of the study. Chickpea varietal samples and data interpretation on varietal differences provided by BT.

## ETHICAL APPROVAL

All procedures involving animals were approved by the Institutional Animal Care Committee (Protocol Number F2012‐035) in accordance with the guidelines of the Canadian Council on Animal Care (Canadian Council on Animal Care, 2018).
